# “I Want It All, and I Want It Now”: Lifetime Prevalence and Reasons for Using and Abstaining from Controlled Performance and Appearance Enhancing Substances (PAES) among Young Exercisers and Amateur Athletes in Five European Countries

**DOI:** 10.3389/fpsyg.2017.00717

**Published:** 2017-05-22

**Authors:** Lambros Lazuras, Vassilis Barkoukis, Andreas Loukovitis, Ralf Brand, Andy Hudson, Luca Mallia, Michalis Michaelides, Milena Muzi, Andrea Petróczi, Arnaldo Zelli

**Affiliations:** ^1^Department of Psychology, Sociology and Politics, Sheffield Hallam University Sheffield, UK; ^2^Department of Physical Education and Sport Sciences, Aristotle University of Thessaloniki Thessaloniki, Greece; ^3^Department of Sport and Exercise Psychology, University of Potsdam Potsdam, Germany; ^4^School of Education, Faculty of Health, Social Care and Education, Kingston University and St George’s University of London London, UK; ^5^Department of Movement, Human and Health Sciences, University of Rome “Foro Italico” Rome, Italy; ^6^Cyprus Sport Organization Nicosia, Cyprus; ^7^School of Life Sciences, Engineering and Computing, Faculty of Science, Engineering and Computing, Kingston University London, UK

**Keywords:** doping, behavioral reasoning, exercise, fitness, recreational sport, young adults

## Abstract

Doping use in recreational sports is an emerging issue that has received limited attention so far in the psychological literature. The present study assessed the lifetime prevalence of controlled performance and appearance enhancing substances (PAES), and used behavioral reasoning theory to identify the reasons for using and for avoiding using controlled PAES in young exercisers across five European countries, in the context of the “SAFE YOU” Project. Participants were 800 young amateur athletes and exercisers (*M* = 21.56; *SD* = 2.69) from Cyprus, Germany, Greece, Italy, and UK who completed an anonymous questionnaire that included measures of self-reported use of controlled PAES, as well as reasons for using and not using controlled PAES. The results of the descriptive analyses demonstrated that almost one out five exercisers in the sample had a previous experience with controlled PAES. Higher prevalence rates were found in Greece and Cyprus and lower in Italy. The most frequently reported reasons for using controlled PAES included achieving the desired results faster; pushing the self to the (physical) limits; and recovering faster after exercise/training. Furthermore, the most frequently reported reasons for not using controlled PAES involved worry about any possible adverse health effects; not feeling the need for using them; and wanting to see what can be achieved naturally without using any controlled PAES. The findings of the present study indicate that the use of controlled PAES is fast becoming a crisis in amateur sports and exercise settings and highlight the need for preventive action and concerted anti-doping education efforts.

## Introduction

Physical activity and exercise participation have been associated with a wide range of benefits to mental and physical health in diverse populations, from healthy young people, to adults suffering from mental illness and elderly people suffering from dementia (de Bruijn et al., 2013; Reiner et al., 2013; Rosenbaum et al., 2014). Physically active people tend to enjoy a higher health-related quality of life (Bize et al., 2007), live longer (Arem et al., 2015), be happier (Wang et al., 2012), and feel more satisfied with their lives (Maher et al., 2015). Furthermore, physical inactivity is considered to be in the top five risk factors for premature morbidity and mortality in developed countries, and has been associated with major non-communicable diseases, including diabetes, coronary heart disease, and several types of cancer (Lee et al., 2012). Scholars and policy-makers have called for greater promotion of physical activity and exercise globally as a means to reduce global early morbidity and mortality rates, and to sustain healthier and more active lifestyles across the lifespan (Reis et al., 2016).

Nonetheless, the health-enhancing properties of physical activity and exercise can be offset by certain behaviors, such as the misuse of performance and appearance enhancement substances (PAES). In leisure time physical activity and recreational sport settings, PAES come in two main forms. Uncontrolled PAES are those substances that can be freely purchased without any restrictions, and with no legal or social sanctions ensuing from their use (i.e., protein formulas, creatine, amino-acids, minerals, and vitamins). On the other hand, controlled PAES are those substances that their use entails legal sanctions (i.e., their use is controlled/regulated by law), are defined as ””doping” substances, and, therefore, are prohibited in elite and competitive sports according to the rules and regulations of the World Anti-Doping Agency (WADA), and commonly represent pharmaceutical compounds and prescribed medication originally used to treat diseases. By this definition, controlled PAES include hormonal substances like anabolic androgenic steroids (AAS) and other testosterone derivatives, growth hormone and insulin-growth factor, erythropoietin, and psychoactive substances like cannabinoids, stimulants, and opioids (Lazuras and Barkoukis, 2014).

### Use of Controlled PAES in Physical Activity Settings

Whereas the use of controlled PAES is highly regulated by sports authorities governing competitive sports and has been considered as a cheating behavior (Morente-Sánchez et al., 2013), the legal and social sanctions associated with the use of controlled PAES in fitness and recreational sports are less stringent. For instance, the 2014 UK Steroid Law specifies that the possession and use of AAS is not an offense (i.e., exercisers who live in the UK can possess and use AAS). At the same time, the UK Anti-Doping clearly states that the presence of AAS compounds and other prohibited PAES or their metabolites in the biological samples of British competitive and/or elite athletes constitutes an Anti-Doping Rule Violation (ADRV) and is followed by severe sanctions according to the WADA anti-doping code. Other countries, like Denmark, apply more stringent policies against the use of controlled PAES in both leisure time physical activity and competitive sport settings (i.e., the same anti-doping laws and sanctions apply inside and outside main Danish sports organization).

Despite existing policies and regulations, the use of controlled PAES has become increasingly popular among non-athletes over the last decade, especially among younger populations. More specifically, Pope et al. (2014) estimated that among Americans aged between 19 and 50 years, about 2.9–4 million people have used AAS at least once in their lifetime, 22% of users have done so before the age of 20, and up to 25% of users have experiences AAS dependence symptoms. Surprisingly, although the non-medical use of controlled PAES, such as AAS, was confined among competitive bodybuilders in the 1960s, it has presently become more common among non-athletes and exercisers with no athletic ambitions who simply want to improve their physical appearance and strength (Parkinson and Evans, 2006; Kanayama et al., 2010). However, the non-medical use of AAS and other controlled PAES has been associated with a wide range of adverse mental and physical health symptoms (e.g., depression, anxiety, mood and body image disturbances, kidney and liver damage, elevated blood pressure), as well as sudden death (Hartgens and Kuipers, 2004; Darke et al., 2014; Frati et al., 2015). The health consequences of controlled PAES use can be even more pronounced among younger (than older) users (Quaglio et al., 2009).

Unlike controlled PAES, the use of uncontrolled PAES (e.g., dietary supplements) has not been extensively associated with adverse and potentially lethal symptoms, except in the case of certain products that were later considered controlled PAES (e.g., ephedrine) or products being contaminated with AAS and other toxic compounds (Geyer et al., 2008; Kohler et al., 2010). Current evidence shows that using dietary supplements is associated with future use of AAS in both adolescent and young adults in physical activity and sports settings (Dodge and Jaccard, 2006; Ntoumanis et al., 2014).

### Behavioral Reasoning Theory and PAES Use

A large body of research has shown that the use of controlled PAES in competitive and elite sport settings is rather as goal-directed and intentional (Lucidi et al., 2008; Lazuras et al., 2015). Albeit manifested in a different (and possibly less competitive) context, the use of controlled PAES in physical activity and exercise settings can also be seen as goal-directed (Allahverdipour et al., 2012; Petróczi, 2013; Ntoumanis et al., 2014). Theories of intention-formation and decision-making processes can help us better understand how athletes and/or exercisers decide to use controlled PAES (Lazuras, 2015). Behavioral reasoning theory is a relatively new model of human decision-making and behavior that aims to better understand the link between beliefs, intentions and behavior by addressing the reasons why people engage (or avoid engaging) in certain behaviors (Westaby, 2005). According to this theory, context-specific reasons play a key role in the intention-formation and behavior-initiation process, and that a better understanding of reasons can elucidate how behavior-specific beliefs (e.g., attitudes) are formed (Westaby et al., 2010). Applying the behavioral reasoning theory in the context of PAES use in exercise and fitness settings can significantly extend the existing knowledge base by revealing the multitude of reasons exercisers decide to use (or to abstain from using) PAES (Petróczi et al., 2017). While a lot of research on controlled PAES has focused on intention-formation theories, such as the theory of reasoned action and the theory of planned behavior (Ntoumanis et al., 2014), a single study applied behavioral reasoning in the context of doping use in elite sports (Overbye et al., 2013), but no study has addressed the role of context-behavioral reasons in the context of controlled PAES use in exercise.

### The Present Study

Millions of young people engage in physical activity and exercise globally, and there are calls for efforts to further increase the number of people engaging in physical activity in the near future as this is expected to yield greater public health benefits, and to reduce early lifestyle-related morbidity and mortality (Lee et al., 2012; Reis et al., 2016). However, the benefits of physical activity can be countered by the use and/or misuse of controlled PAES and the ensuing adverse health effects, especially among younger people (Quaglio et al., 2009). Considering the number of young people currently engaging in physical activity, as well as the projected number of young people who will be exercising in the future, it is of paramount importance to safeguard physical activity and exercise from potentially unhealthy and unsafe practices such as the use of controlled PAES. Doing so requires a better understanding of PAES use patterns and prevalence among young exercisers at an international scale, as well as a better understanding of the reasons why some exercisers decide to engage (or avoid engaging) in the use of controlled PAES. Studying such reasons can further elucidate the underlying decision-making and intention-formation process in the specific context (Westaby, 2005) and inform future anti-doping campaigns. So far, there has been a relative paucity of research on the prevalence of controlled PAES among young exercisers, and the existing studies have focused on single countries. Therefore, a more comprehensive and international perspective on the use of controlled and uncontrolled PAES is warranted. Accordingly, no studies have assessed the behavioral reasons for using and not using controlled PAES among recreational sport participants thus far.

The present study assessed the lifetime prevalence of controlled PAES, as well as the reasons for using and for avoiding using controlled PAES in a large sample of young exercisers across five European countries. The study reported here is part of a larger-scale, European-wide research project (Project SAFE YOU^1^) and was concerned with research and education against doping use among young people in exercise settings. Below, we report findings about the prevalence of controlled and uncontrolled PAES use, demographic characteristics of users, as well as reasons for using and for avoiding PAES use.

## Materials and Methods

### Sample

A total of 800 exercisers participated in the study with an age range between 16 and 25 years old (*M* = 21.56; *SD* = 2.69; males = 499, females = 285; 10 participants preferred not to say or did not report their gender). Participants were recruited from five European countries namely Cyprus, Germany, Greece, Italy, and the UK (see **Table 1** for information on the participants in each country). An analysis of variance (ANOVA) indicated that German participants were significantly older and UK participants were significantly younger than participants from Cyprus, Greece, and Italy, *F*(_4, 799_) = 132.40, *p* < 0.001. Participants were randomly selected and invited to take part in the study either in the gum or through online advertising of the study. Eligible participants should exercise systematically (i.e., 2–3 times per week). The participants were recreational exercisers and amateur athletes mainly involved in fitness, amateur weightlifting, and bodybuilding, as well as team sport (e.g., soccer and basketball).

**Table 1:** Participants’ characteristics in each country.

	*N*	M age	SD	*N* males	*N* females	M years of experience	SD
Cyprus	40	22.32	2.20	25	15	6.45	3.63
Germany	187	23.11	1.83	99	85	9.86	5.80
Greece	196	21.89	2.76	151	45	5.85	4.06
Italy	218	22.20	1.56	128	86	13.13	15.80
UK	159	18.25	2.02	96	54	8.07	4.47

Participants had an average of 9.23 years (*SD* = 9.57) of sport participation experience. An ANOVA indicated that Italian and German participants reported significantly more years of experience as compared to Greek and Cypriot participants, *F*(_4, 778_) = 17.83, *p* < 0.001. In Cyprus and Greece the participants were mainly gym users and exercisers involved in fitness, non-competitive body-building, martial arts and running, and gym/fitness users comprised the sample from Germany. Participants in Italy were university students in sport sciences who involved in physical activity and exercise programs. Finally, the participants in the UK sample were senior high school and university students who regularly engaged in physical activity and exercise.

### Measures

A structured and anonymous questionnaire was used that included measures of self-reported use of controlled PAES, as well as reasons for using and not using controlled PAES.

#### Use of Controlled PAES

To ensure that all participants had the same understanding of controlled PAES, the following definition was given at the beginning of the questionnaire: *“Controlled PAES refer to those substances that are used to build muscles, lose weight and generally enhance the athletic performance but are controlled by the government and/or a sport governing body such as the WADA because they can cause harm. Examples of controlled PAES include anabolic steroids, growth hormones, EPO, stimulants, substances that increase blood flow or open airways. With this definition, we do not include any kind of dietary supplements (e.g., vitamins, proteins, and minerals) that are used to enhance nutrition intake and are not controlled and are available to anyone without restrictions.”* The use of controlled PAES was measured with a single question ‘Which one of the following statements regarding PAES use describes you the best?’ followed by five response options; 1 ( = I currently use PAES and people who are important to me know about it), 2 ( = I currently use PAES but people who are important to me don’t know about it), 3 ( = I used PAES in the past but I do not use now and people who are important to me knew about it), 4 ( = I used PAES in the past but I do not use now and people who are important to me didn’t know about it), and 5 ( = I never used PAES). Participants who marked the fifth option were deemed as *lifetime non-users* of controlled PAES, whereas those marking any of the other response options (i.e., self-reporting controlled PAES use) were deemed *lifetime users* of controlled PAES.

#### Reasons for Using PAES

Participants who self-reported the use of controlled PAES were further asked to complete a questionnaire denoting their reasons for doing so. The items of this questionnaire were generated by the researchers based on the proposed utility of such approach (Petróczi et al., 2017); as well as informal discussions with PAES users and previous literature (Lentillon-Kaestner and Carstairs, 2010; Overbye et al., 2013). The response options included 10 reasons for PAES use (example items ‘it helps to achieve my performance or appearance-related goals,’ and ‘it helps to achieve my desired results faster’). Responses were anchored on a 6-point Likert-type scale ranging from 1 (*not true for me at all*) to 6 (*very true for me*). Cronbach’s α coefficient indicated high and satisfactory internal consistency (α = 0.96).

#### Reasons for Not Using PAES

Participants who did not self-report the use of controlled PAES were asked to complete a questionnaire about their reasons for their abstinence. Similarly to the Reasons for Using PAES questionnaire, the items were generated based on informal discussions with non-users and past research (Lentillon-Kaestner and Carstairs, 2010; Overbye et al., 2013). The response options comprised 12 items (example items: ‘I worry about possible side effects on my health’ and ‘People whose opinion is important to me do not want me to use it’). Participants responded on a 6-point Likert-type scale ranging from 1 (*not true for me at all*) to 6 (*very true for me*). The internal consistency of the scale was high (Cronbach’s α = 0.87).

### Procedure

The study obtained approval by the Faculty Research Ethics Committee, Faculty of Science, Engineering and Computing, Kingston University London. Sport gyms, recreational clubs, student unions, and schools were approached and the aim of the study was described. The collection of the data run in all participating countries: Cyprus, Germany, Greece, Italy, and the UK. The surveys were completed online (in Italy and the UK, part of the sample in Germany) or face-to-face with printed questionnaires (in Cyprus, Greece, and part of the sample in Germany). In the face-to face condition participants completed the surveys alone under the supervision of trained personnel. Completion lasted approximately 20 min. In line with the code of human research ethics of the British Psychological Society, all participants were duly informed about the aims and purposes of the study, provided implied informed consent by voluntary participation, were informed about their participations rights (i.e., voluntary participation, termination of participation at any time without prior notice and negative consequences), and were reassured about the anonymity and confidentiality of their data. For participants younger than 18 years old parental consent was also obtained. The survey was anonymous and participants were re-assured that the surveys will be used for research purposes solely.

### Data Analysis

Statistical analyses were performed with the use of SPSS 22.0. Descriptive statistics were used to estimate the prevalence of controlled PAES use. Differences among the countries in reasons for using or for abstaining from controlled PAES’ use were tested with ANOVA.

## Results

### Lifetime Prevalence of Controlled PAES Use

The prevalence of controlled PAES use for the total sample in each country is reported in **Table 2**. Overall, the analysis indicated that 18.3% of the total sample of participants had some experience with PAES use at least once in their lifetime, either in the past or in the present (i.e., 81.7% declared that they never used controlled PAES). A chi-square test of independence was performed to examine the relation between country and PAES use. The relation between these variables was significant, χ^2^ (16, *N* = 793) = 51.49, *p* < 0.001. Higher prevalence rates were reported for Cyprus (32.5%) and Greece (27%) where participants were gym users and exercisers, whereas lower rates were found in the Italian sample (11.5%) that comprised by university students. In all countries except Greece, the majority of participants who self-reported controlled PAES use also said that people who were important to them knew about it.

**Table 2:** Self-reported use of controlled PAES in five European countries.

	Total sample	Cyprus	Germany	Greece	Italy	UK
	*N* (%)	*N* (%)	*N* (%)	*N* (%)	*N* (%)	*N* (%)
I currently use PAES and people who are important to me know about it	49 (6.2)	5 (12.5)	14 (7.7)	15 (7.7)	4 (1.8)	11 (7.0)
I currently use PAES but people who are important to me don’t know about it	15 (1.9)	-	2 (1.1)	9 (4.6)	1 (0.5)	3 (1.9)
I used PAES in the past but I do not use now and people who are important to me knew about it	39 (4.9)	3 (7.5)	8 (3.8)	8 (4.1)	13 (6.0)	8 (5.1)
I used PAES in the past but I do not use now and people who are important to me didn’t know about it	42 (5.3)	5 (12.5)	8 (4.4)	21 (10.7)	7 (3.2)	1 (0.6)
I never used PAES	648 (81.7)	27 (67.5)	151 (83.0)	143 (73.0)	193 (88.5)	134 (85.4)

### Reasons for Using Controlled PAES

Analysis of variance was used to assess the differences between countries in the mean scores of reasons for using controlled PAES. Accordingly, analysis of frequencies was used to indicate the most common reasons for using controlled PAES. The results showed that there were non-significant differences between countries, *F*(_4, 119_) = 2.02, *p* = 0.096. Furthermore, frequency analysis in the total sample showed that the three most important reasons for using controlled PAES included: achieving the desired results faster; pushing the self to the (physical) limits; and recovering faster after exercise/training. Following what most people are doing was the less frequently reason reported (**Table 3**).

**Table 3:** Self-reported reasons for using controlled PAES in five European countries.

	Total sample	Cyprus	Germany	Greece	Italy	UK
It helps me achieve my performance or appearance-related goals	37.3	54.6	0.0	35.3	41.2	36.4
I follow a recommendation of someone whose opinion is important to me	30.3	45.5	20.2	36.0	23.5	18.2
It helps me achieve my desired results faster	40.8	54.6	55.6	39.2	29.4	31.8
I want to see how far I can push my physical limits	45.7	63.7	29.4	52.9	35.3	38.1
I follow what most people around me are doing	30.3	27.3	41.2	39.2	17.6	27.3
It helps to recover faster after exercise/training	47.9	54.6	35.3	49.0	52.9	45.5
It helps to aid recovery after injury	34.7	63.6	29.4	37.2	35.3	19.0
I want to get advantage in competition	31.9	63.7	11.8	35.3	23.5	27.2
It is a normal part of any serious exercise/training regime	36.1	54.6	29.4	39.2	29.4	31.8
I’m curious to find out if it really works	40.1	45.5	37.5	41.2	41.2	33.3

*The values shown in each column represent the percentage of participants who scored on the extreme ends of the measurement scale (i.e., 5 = true for me and 6 = very true for me).*

However, each country seemed to have a different profile with respect to the reasons that emerged for using controlled PAES. More specifically, in Cyprus participants reported recovery after injury, having advantage in competition, and pushing the self to its physical limits as the most common reasons for using controlled PAES. On the other hand, following what most people is doing was the less frequently reason reported by the Cypriot participants. In Germany, achieving the desired results faster, following what most people are doing, and curiosity about the effects of PAES on performance and physical appearance were the most frequently reported reasons for using controlled PAES, whereas achieving performance or appearance-related goals was the least frequently reported. Greek exercisers reported pushing their physical limits, recovering faster after exercise/training, and curiosity as the reasons that better described their PAES use, whereas achieving performance or appearance-related goals was the least frequently reported one. In Italy, achieving performance or appearance-related goals, recovering faster after exercise/training, and curiosity were the most frequently reported reasons for PAES use, whilst following what most people is doing was the least frequently reported reason. Finally, in the UK, the most commonly reported reasons for using controlled PAES included achieving performance or appearance-related goals, pushing physical limits, and recovering faster after exercise/training, and following recommendations from important others was the least frequently reported one.

### Reasons for Avoiding Using Controlled PAES

Analysis of variance was also used to assess for between-country differences in the reasons for abstaining from the use of controlled PAES. The results showed that there were non-significant differences in the mean scores between countries, *F*(_4, 540_) = 1.17, *p* = 0.321. Analysis of frequencies further showed that, in the total sample, the most commonly reported reasons for *not using* controlled PAES included: worry about any possible adverse health effects; not feeling the need for using them; and wanting to see what can be achieved naturally without using any controlled PAES. The least frequently reported reason was not being able to afford buying controlled PAES. The results from the frequency analysis concerning the reasons for abstaining from PAES use are reported in **Table 4**.

**Table 4:** Self-reported reasons for not using controlled PAES in five European countries.

	Total sample	Cyprus	Germany	Greece	Italy	UK
I worry about possible side effects on my health	71.2	85.7	85.9	55.5	78.7	60.2
I lack trust in the quality and ingredients	62.8	85.7	73.9	48.9	61.5	60.3
I do not feel the need for it	75.3	85.7	88.7	55.1	80.0	66.7
People whose opinion is important to me do not want me to use it	48.0	50.0	50.0	45.5	48.9	45.7
I worry about the legal consequences	43.4	42.9	48.6	37.5	39.6	47.4
I want to see what I can do naturally	73.0	50.0	74.6	58	79.4	73.9
Not many people around me using it	46.8	14.3	47.5	36.3	50.0	50.1
I cannot afford it	23.3	57.2	19.9	39.1	13.8	28.7
If I use PAES, it is no longer 100% me	68.2	55.5	70.9	58	74.0	64.3
I do not know where to buy it	30.5	20.0	32.9	30.7	22.1	41.7
It would give me unfair advantage in a competition	64.8	77.7	73.0	45.5	69.6	60.7
I do not know what to take and how to take it	37.6	33.3	34.5	38.6	35.6	46.0

*The values shown in each column represent the percentage of participants who scored on the extreme ends of the measurement scale (i.e., 5 = true for me and 6 = very true for me).*

In each country, different reasons emerged as most and least important for abstaining from PAES use. More specifically, in Cyprus, the most commonly reported reason for not using PAES included worry about possible health side effects; not feeling the need for it; and lack of trust in the quality and ingredients of controlled PAES use. The least frequently reported reason was the belief that the majority of people around (i.e., other exercisers) were not using controlled PAES. In Germany and Italy, the most common reasons for not using PAES included worry about possible health side effects, not feeling the need for it, and wanting to see what can be achieved naturally without using controlled PAES. Also, German participants reported not being able to afford buying PAES as the least reported reason, whereas not having access to PAES was the least frequently reported reason in Italy. In Greece and the UK, not feeling the need for it, a not being “100% me” approach, and wanting to see what can be achieved naturally were the most commonly reported reasons for not using controlled PAES. Not having access to PAES and not being able to afford it were the least reported reasons in both Greece and the UK.

## Discussion

The present study assessed the self-reported use of controlled PAES, as well as the reasons for using and for abstaining from PAES use in a large sample of young people involved in exercise and amateur sports across five European countries: Cyprus, Germany, Greece, Italy, and the UK. The results showed that, overall, 18.3% of participants appear to have some sort of experience with the use of controlled PAES, either in the past or in the present. Given that self-reported measures of controlled PAES use may be susceptible to under-reporting biases (Gucciardi et al., 2010; Petróczi et al., 2010; de Hon et al., 2015), the prevalence of PAES use found in the present paper may actually be an underestimation of the actual prevalence of controlled PAES use among young exercisers in the countries we examined. Furthermore, our findings also showed that relatively higher use of controlled PAES was self-reported in Cyprus, Greece, and Germany where participants were mostly gym users and fitness exercisers, than in Italy and UK who were mostly high school and university students. These findings suggest that almost one out of five young exercisers in the overall, European-wide sample that was used in the present study admitted the use of controlled PAES. Importantly, controlled PAES use reflects the misuse of mostly pharmaceutical compounds and prescribed medication, without any medical cause and with the sole motive to enhance physical appearance and athletic performance in physical activity settings. Controlled PAES use has been associated with a wide range of adverse mental and physical health effects, as well as early and sudden death in certain occasions (e.g., Quaglio et al., 2009; Darke et al., 2014; Frati et al., 2015). Apparently, using controlled PAES represents an unsafe practice that can offset the health benefits of physical activity and exercise. Given the current numbers of young people presently involved in exercise and physical activity, as well as the projected number of young exercisers in the next decade, it seems that the use of controlled PAES is an emerging public health concern that merits the attention of both policy-makers and researchers involved in the promotion of physical activity and its health-enhancing properties.

Following from the study by Overbye et al. (2013) about reasons for doping use in elite athletes, our research was the first one to apply concepts from behavioral reasoning theory (Westaby, 2005) to the study of controlled PAES use in an international sample of young exercisers. Interesting findings emerged with respect to the reasons for both using and avoiding using controlled PAES. Overall, it appears that controlled PAES use in the present sample was motivated mostly by the need to achieve the desired goals, whether this is relevant to enhancing athletic performance, improving physical appearance, or both. These findings attest to the goal-directedness of controlled PAES use among young exercisers. The findings also indicate that, albeit most of our participants were exercisers and non-competitive amateur athletes, those who self-reported the use controlled PAES appear to have a “competitive sports’ mindset by being focused too much on improving performance, reaching goals and recovering from training, than enjoying the health benefits of exercise. Curiosity also emerged as a commonly reported reason for using controlled PAES in Italy, Greece, and Germany but not as much in the UK and Cyprus. This finding suggests that preventive action against the use of controlled PAES in exercise settings may benefit by shifting the focus from immediate performance enhancement effects and raising exercisers’ awareness about the actual and long-term effects of controlled PAES use on their mind and body. Similar studies in the context of competitive sports have shown that elite athlete tend to downplay the long-term and harmful effects of doping use on their health by focusing on the immediate effects of doping in performance improvement (e.g., Lentillon-Kaestner et al., 2012). A similar process may be in place in PAES use in amateur and fitness sports, and future studies should further look into risk perception of PAES use in this context.

More specifically, worry about the health effects of controlled PAES use was among the most commonly reported reasons for not using such substances. Other frequently reported abstinence reasons included not feeling the need to use controlled PAES as well as a determination to enjoy exercise and anticipate benefits without using PAES. Possibly, there may be a mentality aspect there with some exercisers being committed to more “natural” and drug-free exercise styles, but this is a tentative argument that requires further empirical investigation. According to behavioral reasoning theory, reasons for and against the use of controlled PAES, may independently predict attitudes and intentions to use PAES (Petróczi et al., 2017). In line with this argument, our findings indicate that reasons for using and for abstaining using controlled PAES differentiate, with the former depicting high achievement motives and a “competitive” mindset, and the latter representing more “natural exercising” motives. Future research may indicate whether this differentiation is reflected in differential attitudes and intentions toward using (and not using) controlled PAES, respectively.

Finally, in Greece and the UK the reasons for not using controlled PAES depicted identity concerns (e.g., “If I use PAES, it is no longer 100% me”), and this indicates that self-identity and/or self-categorization processes may be relevant to the ways exercisers reason and decide to act with respect to the use of controlled PAES. Rees et al. (2015) recently argued that a social identity approach can broadly benefit research on sport and exercise psychology issues. Nevertheless, no study so far has addressed social identity and/or self-categorization in the context of controlled PAES use either in competitive/elite sports or in exercise/amateur sports settings.

Furthermore, our findings can also be explained in the context of other theoretical approaches than behavioral reasoning theory. More specifically, the seeming urgency for immediate performance improvement (e.g., achieving desired results and/or recovering from injury faster) that surfaced in our analysis of the reasons for using PAES indicates that impulsivity/urgency and self-regulation failure may partly explain the psychological process underlying PAES use in amateur and fitness sports. Perhaps the application of self-regulation theory, such as Metcalfe and Mischel’s (1999) analysis of self-regulation failure and delayed gratification, may add further to our understanding of PAES use in this population. Incorporating self-regulatory capacities in the study of PAES use in amateur sports will also further extend existing social cognitive models of doping use in competitive sports (e.g., Chan et al., 2015).

Overall, the present study showed that roughly one out of five young exercisers self-reported the use of controlled PAES Preventive action is needed in order to control the use of PAES in physical activity and exercise settings; otherwise, the health-enhancing properties of exercising may be compromised. Furthermore, a competitive “mindset” appears to dominate the reasons for using controlled PAES, with a strong emphasis on improving performance and achieving results faster. On the contrary, worry about adverse health effects and a commitment to being natural and unaffected by PAES use dominated the reasons for abstaining from controlled PAES use. While providing insightful findings about PAES use in amateur and fitness sports, our study was not free of limitations. In particular, our findings relied on self-reports and, therefore, may be influenced by reporting bias and social desirability (Gucciardi et al., 2010). Therefore, the prevalence rates we report in this paper may actually underestimate the actual prevalence of controlled PAES in our population. Secondly, we did not account for the co-occurrence of controlled and uncontrolled PAES (e.g., nutritional supplements). This would allow us to provide more detailed analysis of PAES use patterns, by identifying exercisers/amateur athletes that were non-users, those who used only uncontrolled PAES (e.g., nutritional supplements), and those who used both nutritional supplements and controlled PAES, or only controlled PAES. Such an analysis would be meaningful to identify if users of nutritional supplements display similar motivational patterns with PAES users with respect to the reasons for using (or avoiding the use of) controlled PAES. Past research has shown that users of nutritional supplements tend to view doping substances more favorably than non-users of supplements (Barkoukis et al., 2015). Future research may further address those limitations and provide further insights into the psychological processes underlying PAES use in amateur and recreational sports.

## Ethics Statement

This study was carried out in accordance with the recommendations of ‘Faculty Research Ethics Committee, Faculty of Science, Engineering and Computing, Kingston University London’ with written informed consent from all subjects. All subjects gave written informed consent in accordance with the Declaration of Helsinki. The protocol was approved by the ‘Faculty Research Ethics Committee, Faculty of Science, Engineering and Computing, Kingston University London.’

## Author Contributions

All authors listed, have made substantial, direct and intellectual contribution to the work, and approved it for publication.

## Conflict of Interest Statement

The authors declare that the research was conducted in the absence of any commercial or financial relationships that could be construed as a potential conflict of interest.

## Footnotes

1 http://safeyou.eu/
