# Corrigendum: “I Want It All, and I Want It Now”: Lifetime Prevalence and Reasons for Using and Abstaining from Controlled Performance and Appearance Enhancing Substances (PAES) among Young Exercisers and Amateur Athletes in Five European Countries

**DOI:** 10.3389/fpsyg.2018.01162

**Published:** 2018-07-25

**Authors:** Lambros Lazuras, Vassilis Barkoukis, Andreas Loukovitis, Ralf Brand, Andy Hudson, Luca Mallia, Michalis Michaelides, Milena Muzi, Andrea Petróczi, Arnaldo Zelli

**Affiliations:** ^1^Department of Psychology, Sociology and Politics, Sheffield Hallam University, Sheffield, United Kingdom; ^2^Department of Physical Education and Sport Sciences, Aristotle University of Thessaloniki, Thessaloniki, Greece; ^3^Department of Sport and Exercise Psychology, University of Potsdam, Potsdam, Germany; ^4^School of Education, Faculty of Health, Social Care and Education, Kingston University and St George's University of London, London, United Kingdom; ^5^Department of Movement, Human and Health Sciences, University of Rome “Foro Italico”, Rome, Italy; ^6^Cyprus Sport Organization, Nicosia, Cyprus; ^7^School of Life Sciences, Engineering and Computing, Faculty of Science, Engineering and Computing, Kingston University, London, United Kingdom

**Keywords:** doping, behavioral reasoning, exercise, fitness, recreational sport, young adults

Due to a data processing error, a single case from the Greek sample and cases from the Cypriot sample were removed. The re-analysis of the data without these cases resulted in minor changes in all tables and a few sentences in the manuscript. These changes do not influence the scientific conclusions of the article.

In the original article, there was a mistake in Table 1 as published. Due to adjusted sub-sample sizes for Cyprus and Greece, figures have been marginally changed. The corrected Table 1 appears below.

**Table 1:** Participants characteristics in each country.

	***N***	**M age**	**SD**	***N* males**	***N* females**	**M years of experience**	**SD**
Cyprus	40	22.32	2.20	25	15	6.45	3.63
Germany	187	23.11	1.83	99	85	9.86	5.80
Greece	196	21.89	2.76	151	45	5.85	4.06
Italy	218	22.20	1.56	128	86	13.13	15.80
UK	159	18.25	2.02	96	54	8.07	4.47

In the original article, there was a mistake in Table 2 as published. Due to adjusted sub-sample sizes for Cyprus and Greece, figures have been marginally changed. The corrected Table 2 appears below.

**Table 2:** Self-reported use of controlled PAES in five European countries.

	**Total sample *N* (%)**	**Cyprus *N* (%)**	**Germany *N* (%)**	**Greece *N* (%)**	**Italy *N* (%)**	**UK *N* (%)**
I currently use PAES and people who are important to me know about it	49 (6.2)	5 (12.5)	14 (7.7)	15 (7.7)	4 (1.8)	11 (7.0)
I currently use PAES but people who are important to me don't know about it	15 (1.9)	–	2 (1.1)	9 (4.6)	1 (0.5)	3 (1.9)
I used PAES in the past but I do not use now and people who are important to me knew about it	39 (4.9)	3 (7.5)	8 (3.8)	8 (4.1)	13 (6.0)	8 (5.1)
I used PAES in the past but I do not use now and people who are important to me didn't know about it	42 (5.3)	5 (12.5)	8 (4.4)	21 (10.7)	7 (3.2)	1 (0.6)
I never used PAES	648 (81.7)	27 (67.5)	151 (83.0)	143 (73.0)	193 (88.5)	134 (85.4)

In the original article, there was a mistake in Table 3 as published. Due to adjusted sub-sample sizes for Cyprus and Greece, figures have been marginally changed. The corrected Table 3 appears below.

**Table 3:** Self-reported reasons for using controlled PAES in Five European Countries.

	**Total sample**	**Cyprus**	**Germany**	**Greece**	**Italy**	**UK**
It helps me achieve my performance or appearance-related goals	37.3	54.6	0.0	35.3	41.2	36.4
I follow a recommendation of someone whose opinion is important to me	30.3	45.5	20.2	36.0	23.5	18.2
It helps me achieve my desired results faster	40.8	54.6	55.6	39.2	29.4	31.8
I want to see how far I can push my physical limits	45.7	63.7	29.4	52.9	35.3	38.1
I follow what most people around me are doing	30.3	27.3	41.2	39.2	17.6	27.3
It helps to recover faster after exercise/training	47.9	54.6	35.3	49.0	52.9	45.5
It helps to aid recovery after injury	34.7	63.6	29.4	37.2	35.3	19.0
I want to get advantage in competition	31.9	63.7	11.8	35.3	23.5	27.2
It is a normal part of any serious exercise/training regime	36.1	54.6	29.4	39.2	29.4	31.8
I'm curious to find out if it really works	40.1	45.5	37.5	41.2	41.2	33.3

*The values shown in each column represent the percentage of participants who scored on the extreme ends of the measurement scale (i.e., 5 = true for me, and 6 = very true for me)*.

In the original article, there was a mistake in Table 4 as published. Due to adjusted sub-sample sizes for Cyprus and Greece, figures have been marginally changed. The corrected Table 4 appears below.

**Table 4:** Self-reported reasons for not using controlled PAES in Five European Countries.

	**Total sample**	**Cyprus**	**Germany**	**Greece**	**Italy**	**UK**
I worry about possible side effects on my health	71.2	85.7	85.9	55.5	78.7	60.2
I lack trust in the quality and ingredients	62.8	85.7	73.9	48.9	61.5	60.3
I do not feel the need for it	75.3	85.7	88.7	55.1	80.0	66.7
People whose opinion is important to me do not want me to use it	48.0	50.0	50.0	45.5	48.9	45.7
I worry about the legal consequences	43.4	42.9	48.6	37.5	39.6	47.4
I want to see what I can do naturally	73.0	50.0	74.6	58	79.4	73.9
Not many people around me using it	46.8	14.3	47.5	36.3	50.0	50.1
I cannot afford it	23.3	57.2	19.9	39.1	13.8	28.7
If I use PAES, it is no longer 100% me	68.2	55.5	70.9	58	74.0	64.3
I do not know where to buy it	30.5	20.0	32.9	30.7	22.1	41.7
It would give me unfair advantage in a competition	64.8	77.7	73.0	45.5	69.6	60.7
I do not know what to take and how to take it	37.6	33.3	34.5	38.6	35.6	46.0

*The values shown in each column represent the percentage of participants who scored on the extreme ends of the measurement scale (i.e., 5 = true for me, and 6 = very true for me)*.

The authors apologize for these errors and state that they do not change the scientific conclusions of the article in any way.

## Text correction

1. In the Abstract, it is stated “Participants were 915 young amateur athletes and exercisers (*M* = 21.62; *SD* = 2.62) from Cyprus, Germany, Greece, Italy, and UK who completed an anonymous questionnaire that included measures of self-reported use of controlled PAES, as well as reasons for using and not using controlled PAES.”
2. In the Materials and Methods section, Sample, it is stated that “A total of 915 exercisers participated in the study with an age range between 16 and 25 years old (*M* = 21.62; *SD* = 2.62; males = 584, females = 315; 16 participants preferred not to say or did not report their gender)”. It is also stated that “Participants had an average of 8.85 (*SD* = 9.06) years of sport participation experience.”
  In the same section and paragraph, the reported *F*-value for age differences is “[*F*_(4, 914)_ = 135.03, *p* < 0.001]”, and for differences in years of sport experience is” [*F*_(4, 891)_ = 22.92, *p* < 0.001].”
3. In the Results section, Lifetime Prevalence of Controlled PAES Use, it is stated that “19.3% of the total sample of participants had some experience with PAES use at least once in their lifetime, either in the past or in the present (i.e., 80.7% declared that they never used controlled PAES).”
  In the same paragraph it is also stated that “The relation between these variables was significant, χ^2^ (16, *N* = 908) = 62.85, *p* < 0.001. Higher prevalence rates were reported for Cyprus (28.9%) and Greece (27.6%).”
4. In the Results section, in Reasons for Using Controlled PAES, one of the most frequently reported reasons for avoiding using controlled PAES is incorrect. Specifically, it is stated that “in Cyprus participants reported recovery after injury, having advantage in competition, and being a normal part of any serious exercise/training regime as the most common reasons for using controlled PAES.”
5. In the Results section, Reasons for Using Controlled PAES, the reported statistical values in the following sentence are incorrect “[*F*_(4, 575)_ = 1.15, *p* = 0.318].”
  On the same section, in Reasons for Avoiding Using Controlled PAES, the reported statistical values in the following sentence are incorrect “*F*_(4, 575)_ = 1.15, *p* = 0.318]”
6. In the Discussion section, first paragraph, it is stated that “overall, 19.3% of participants appear to have some sort of experience with the use of controlled PAES.” This statistical value is incorrect.

**The following corrections have been made to:**

1. In the Abstract, the corrected sample characteristics are presented:
  “Participants were 800 young amateur athletes and exercisers (*M* = 21.56; *SD* = 2.69) from Cyprus, Germany, Greece, Italy, and UK who completed an anonymous questionnaire that included measures of self-reported use of controlled PAES, as well as reasons for using and not using controlled PAES.”
2. In the Materials and Methods section, Sample, the corrected sample characteristics are presented:
  “A total of 800 exercisers participated in the study with an age range between 16 and 25 years old (*M* = 21.56; *SD* = 2.69; males = 499, females = 285; 10 participants preferred not to say or did not report their gender).”
  “Participants had an average of 9.23 years (*SD* = 9.57) of sport participation experience.”
  The revised *F*-values are also reported for differences in mean age of participants: “[*F*_(4, 799)_ = 132.40, *p* < 0.001],” and for differences in mean years of sport participation experience “[F_(4, 778)_ = 17.83, *p* < 0.001].”
3. In the Results section, sub-section Lifetime Prevalence of Controlled PAES Use, 1st paragraph:
  “18.3% of the total sample of participants had some experience with PAES use at least once in their lifetime, either in the past or in the present (i.e., 81.7% declared that they never used controlled PAES).”
  “The relation between these variables was significant, χ^2^ (16, *N* = 793) = 51.49, *p* < 0.001. Higher prevalence rates were reported for Cyprus (32.5%) and Greece (27%).”
4. In the Results section, in Reasons for Using Controlled PAES:
  “in Cyprus participants reported recovery after injury, having advantage in competition, and pushing the self to its physical limits as the most common reasons for using controlled PAES.”
5. In the Results section, sub-section Reasons for Using Controlled PAES, the correct values have been added:
  “[*F*_(4, 119)_ = 2.02, *p* = 0.096].”
  In the Results section, sub-section Reasons for Avoiding Using Controlled PAES, the correct values have been added:
  “[*F*_(4, 540)_ = 1.17, *p* = 0.321].”
6. In the Discussion section, first paragraph, the correct statistical value is reported:
  “overall, 18.3% of participants appear to have some sort of experience with the use of controlled PAES.”
  The authors apologize for these errors and the inconvenience it might have caused.
  The original article has been updated.

### Conflict of interest statement

The authors declare that the research was conducted in the absence of any commercial or financial relationships that could be construed as a potential conflict of interest.

